# Redescription of *Clinostomum phalacrocoracis* metacercariae (Digenea: Clinostomidae) in cichlids from Lake Kinneret, Israel

**DOI:** 10.1051/parasite/2014034

**Published:** 2014-07-03

**Authors:** Monica Caffara, Nadav Davidovich, Rama Falk, Margarita Smirnov, Tamir Ofek, David Cummings, Andrea Gustinelli, Maria L. Fioravanti

**Affiliations:** 1 Department of Veterinary Medical Sciences, Alma Mater Studiorum University of Bologna Via Tolara di Sopra 50 40064 Ozzano Emilia (BO) Italy; 2 Central Fish Health Laboratory, Department of Fisheries and Aquaculture, Ministry of Agriculture and Rural Development 10803 Nir David Israel; 3 Israel Oceanographic and Limnological Research, Kinneret Limnological Laboratory 14950 Migdal Israel

**Keywords:** *Clinostomum phalacrocoracis*, Metacercaria, Wild Cichlids, Lake Kinneret, Israel, Molecular Analysis

## Abstract

Clinostomidae are digeneans characterized by a complex taxonomic history, continuously under revision based on both morphological and molecular analysis. Among the 14 species considered valid so far *Clinostomum phalacrocoracis* has been well described only at the adult stage, whereas the morphology of the metacercarial stage has been reported only once. During a parasitological survey carried out on 262 wild cichlids sampled from Lake Kinneret (Israel) metacercariae referable to *C. phalacrocoracis* were found in 18 fingerlings. In this study, we report this clinostomid species for the first time in wild fish from Israel describing the metacercarial stage of *Clinostomum phalacrocoracis*, coupling its morphological description with molecular analysis carried out on ITS rDNA and COI mtDNA sequences.

## Abstract (fr)

Les Clinostomidae sont des digènes caractérisés par une histoire taxonomique complexe, constamment en cours de révision sur la base d’analyses morphologiques et moléculaires. Parmi les 14 espèces considérées comme valides, *Clinostomum phalacrocoracis* n’a été bien décrit qu’au stade adulte alors que la morphologie de la métacercaire n’a été rapportée qu’une seule fois. Pendant une enquête parasitologique réalisée sur 262 cichlidés sauvages prélevés dans le Lac de Tibériade (Israël), des métacercaires rapportées à *C. phalacrocoracis* ont été trouvées dans 18 poissons. Dans cette étude, nous présentons pour la première fois cette espèce de Clinostomidae chez les poissons sauvages en provenance d’Israël et redécrivons la métacercaire de *Clinostomum phalacrocoracis*, en associant sa description morphologique à une analyse moléculaire réalisée sur des séquences ITS (ADNr) et COI (ADNmt).

## Introduction

Clinostomidae are digenetic trematodes with a heteroxenous life cycle, involving both vertebrate and invertebrate hosts, and have a complex taxonomic history, having been continuously under revision based on both morphological and, more recently, molecular analysis.

Among the 14 *Clinostomum* species considered valid so far [3, 8, 11, 20], only a few are supported by a complete morphological description and most of them are reported only at the adult stage, while the metacercarial phase hosted by fish or amphibians and known as “yellow grub” is often poorly described. *C. phalacrocoracis* has been well described at the adult stage by Dubois [4] and Ukoli [26], but only Kabunda and Sommerville [10] provided a detailed morphological description of one metacercaria of *Clinostomum* sp. referable to *C. phalacrocoracis*. In this study, we redescribe metacercariae of *C. phalacrocoracis* from wild cichlids caught in Lake Kinneret (Israel), coupling the morphological description with molecular analysis.

Lake Kinneret (Sea of Galilee, Lake Tiberias) is the only freshwater lake in Israel, located in the central part of the Jordan rift valley [21], with greater fish abundance at the lake periphery than in its pelagic zone [13]. Three species of *Clinostomum* have been reported in fish from Lake Kinneret so far: (1) *C. complanatum* was found in the muscles of wild Cypriniformes (*Barbus canis, Cyprinus carpio, Capoeta damascina*) and in *Oreochromis niloticus* (formerly *Tilapia nilotica*) farmed in ponds on the lake shores [7, 15]. (2) *C. cutaneum* was detected in subcutaneous and muscle tissues of *Tilapia zilli*, *Tristramella simonis*, *Oreochromis niloticus*, *Sarotherodon galilaeus* and *Astatotilapia flavijosephii* [7, 16]. (3) *C. tilapiae* cysts were observed in the gill arches, muscles and in the internal part of the sclera of several cichlid species [7, 27]. Dzikowski et al. [6] confirmed the presence of *C. complanatum* by molecular analysis. In fact, the molecular approach has contributed to the understanding of the species distribution, but molecular data are available so far only for a limited number of *Clinostomum* species from a few biogeographic regions. Currently, analyses of mitochondrial and/or ribosomal DNA have confirmed the validity of five species: *C. complanatum*, *C. marginatum*, *C. cutaneum*, *C. phalacrocoracis* and *C. tataxumui* [3, 6, 8, 20].

This research reports for the first time the presence of metacercariae of *Clinostomum phalacrocoracis* in wild cichlids collected off the shores of Lake Kinneret during 2012, providing a morphological description and molecular data.

## Materials and methods

### Sampling sites

**Figure 1.**
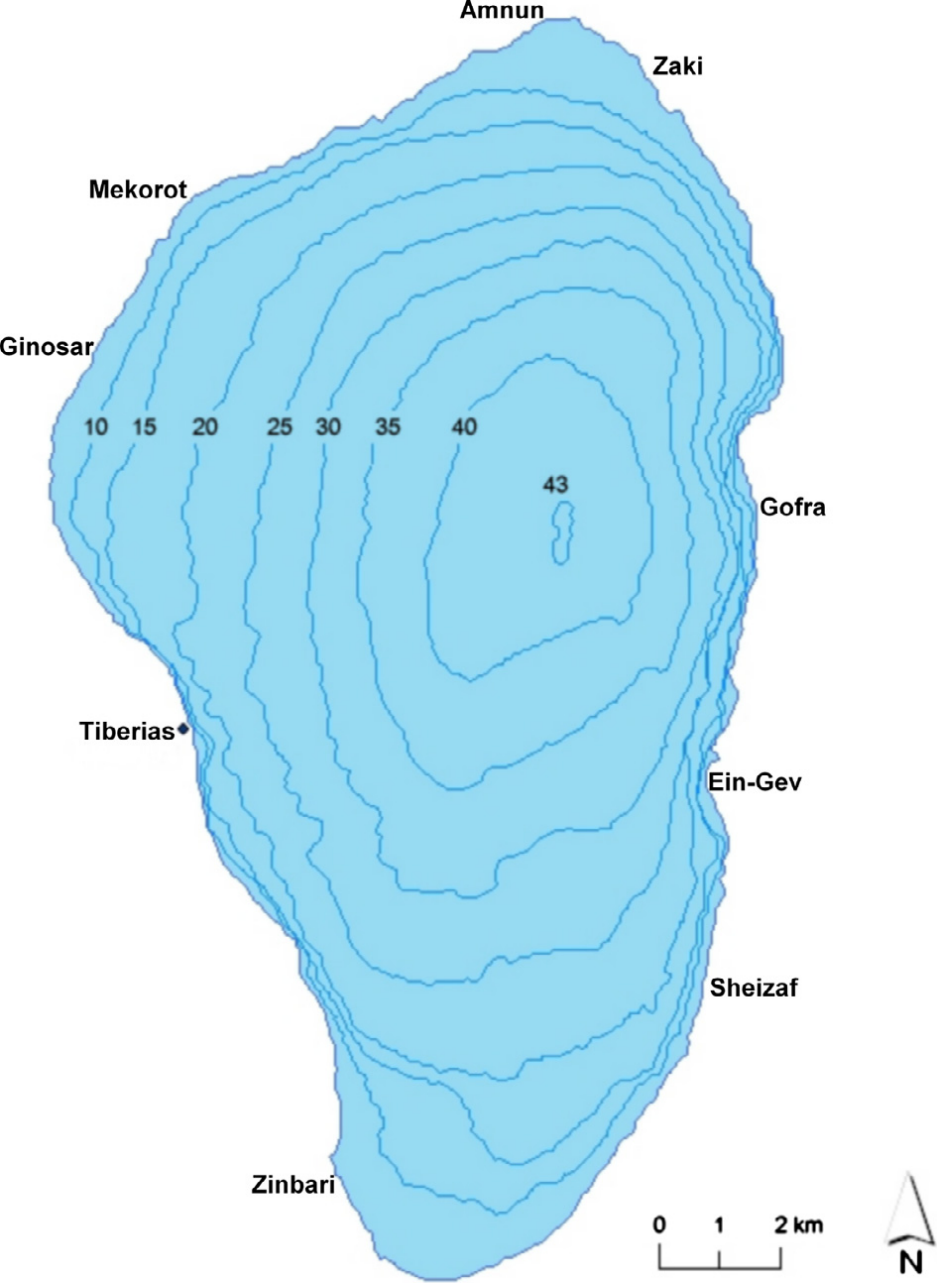
Lake Kinneret map: sampling sites.

Eight different sites along the Lake Kinneret shores (between 32°42′15″ N, 32°53′44″ N and 35°30′52″ E, 35°38′55″ E) were sampled in 2012: Amnun and Zaki (north), Ein-Gev, Gofra and Sheizaf (east), Ginosar and Mekorot (north-west) and Zinbari (south-west) (Fig. 1). Samples were collected from the littoral zone of the lake (less than 10 m depth).

A total of 262 cichlids were caught: 185 adults (113 *Sarotherodon galilaeus*, 32 *Oreochromis aureus*, 40 *Tilapia zillii*) and 77 cichlid fingerlings, unidentifiable to the species level. The fish, collected by a scuba diver and traps of 40 mm × 40 mm plastic mesh, were kept alive until examination at the laboratory where weight, total length and visual inspection to detect macroscopically visible parasites were carried out. Wet preparations of the gills, brain, eyes, kidney, liver, intestine, spleen, fins and gonads were examined under a dissecting and light microscope. All parasites were preserved in 70% ethanol for both morphological and molecular analysis.

### Morphological study

Morphological studies were performed on 13 metacercariae; whole mounts were prepared by clarifying the parasites in Amman’s lactophenol and two specimens were also stained by Malzacher’s method [19]. Line drawings were made with the aid of a drawing tube, and measurements are given in micrometers unless otherwise stated. Measurements were taken following Matthews and Cribb [11]. For molecular analysis, a little piece of the posterior third was cut after measurement of total length and maximum width in 8 out of 13 metacercariae.

### Molecular analysis

Total DNA was extracted using a PureLink Genomic DNA Kit (Invitrogen) following the manufacturer’s protocol. Internal Transcribed Spacer (ITS) rDNA was amplified as reported by Gustinelli et al. [8], while a fragment of *cytochrome c oxidase* I (COI mtDNA) was amplified with the protocol of Moszczynska et al. [12]. PCR products were resolved on a 1% agarose gel stained with SYBR Safe DNA Gel Stain in 0.5X TBE (Molecular Probes – Life Technologies).

For sequencing of both ITS and COI, the bands were excised and purified by NucleoSpin Gel and PCR Clean-up (Mackerey-Nagel) and sequenced with an ABI 3730 DNA analyzer at StarSEQ GmbH (Mainz, Germany). Sequence assembly was carried out using Vector NTI Advance^TM^ 11 software (Invitrogen) and multiple sequence alignments were constructed by BioEdit 7.2. Pairwise distances using the Kimura 2-parameter model and Maximum Likelihood (ML) tree (GTR, 1000 replicate) were calculated by MEGA 5.05 [24].

## Results

**Figure 2.**
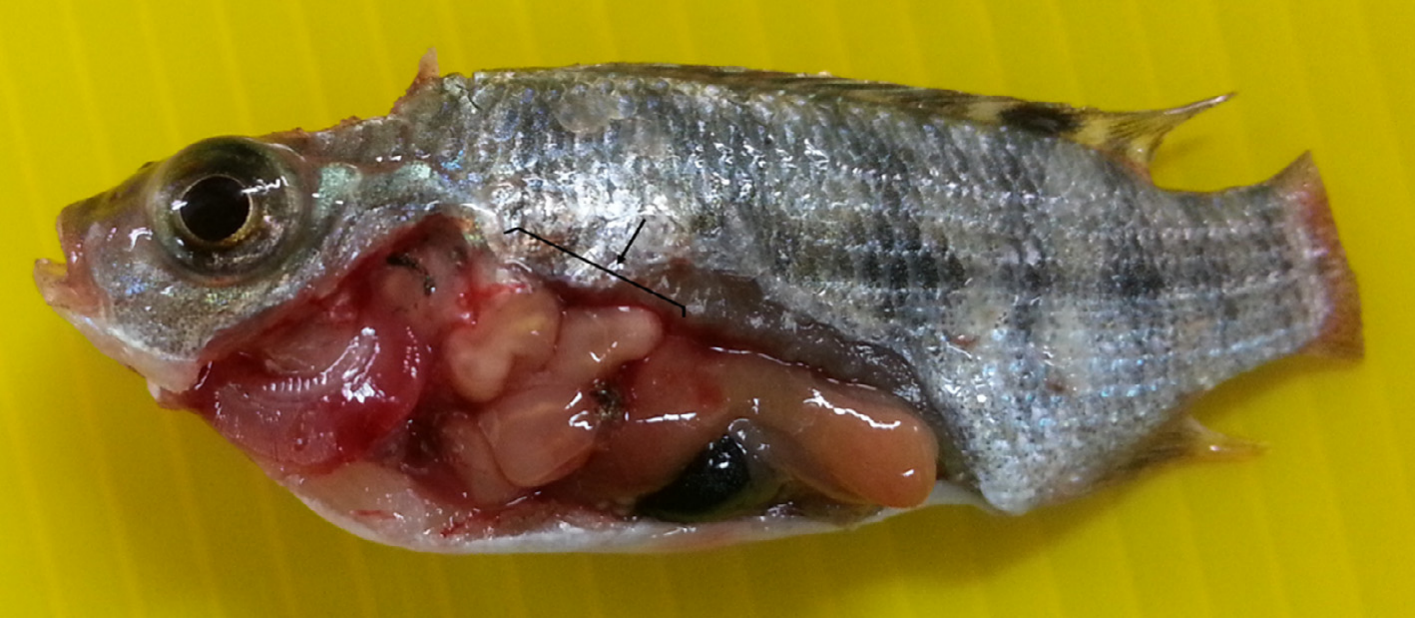
*Clinostomum phalacrocoracis* metacercariae in the body cavity of a cichlid fingerling.

The prevalence of *Clinostomum* spp. metacercariae in cichlid fingerlings was 23.4% (18/77). Infected fish originated from the Mekorot shore (15 fish out of 38 examined) and the Zaki shore (3 fish out of 6). Most of the *Clinostomum* spp. metacercariae were collected from the anterior part of the abdominal cavity (Fig. 2) and in a few cases from the muscles of the gill arches. The average number of parasites collected was 5.9 and the highest number of metacercariae collected from a single fish (weight 4.05 g and total length 5.5 cm) was 22. No *Clinostomum* spp. metacercariae were found in adult fish. All the *Clinostomum* metacercariae examined were identified as *C. phalacrocoracis*.

### *Clinostomum phalacrocoracis* Dubois, 1930 (Fig. 3)

The main morphological characters of the metacercariae were (min-max (mean ± SD) μm): body stout, slightly wider in gonadic region; 9500–15200 (12061 ± 1820) long, 1855–3967 (3075 ± 591.9) wide. Oral sucker, 410–533 (460.4 ± 35.03) long × 478–732 (576.1 ± 83.07) wide, smaller than *acetabulum*, 926–1253 (1094 ± 89.86) long × 1011–1346 (1193.3 ± 105.22) wide, surrounded by weakly developed oral collar. Pharynx evident; intestine bifurcates immediately posterior to pharynx. Intestinal caeca run laterally to ventral sucker and immature genital complex, with small diverticula more evident posteriorly to ventral sucker. Connection between intestinal caeca and excretory system not clearly visible. Testes arranged in tandem between middle and posterior third of body. Anterior testis, 677–1466 (1074.2 ± 255.29) long × 643–1469 (1063.7 ± 266.98) wide, in posterior part of middle third of body, fan-shaped, consists of four to eight blunt lobes, some of which are sub-lobed; sometimes, right lobe slightly displaced to left by uterus. Posterior testis, 606–1182 (957.2 ± 211.70) long × 695–1469 (1072 ± 195.71) wide, in anterior part of posterior third of body, fan-shaped with anterior margin concave and with two major lateral lobes and one posterior lobe, each of which is sub-lobed. Cirrus sac 389–717 (563.5 ± 117.67) long × 143–292 (253.2 ± 44.25) wide, bean-shaped, in dextral intertesticular space, anterior to ovary, with genital pore opening laterally at posterior margin of anterior testis between right and posterior lobe. Genital opening with evident small blunt tubercles along its internal edge. Ovary, 119–378 (278.7 ± 74.07) long × 121–363 (261.9 ± 69.85) wide, irregular, round, smaller than cirrus sac, located in dextral intertesticular space. Uterus runs straight from ventral sucker to anterior testis. Uteroduct runs around left margin of anterior testis and opens into uterine sac. Metraterm, straight and overlapping the right half of anterior testis, connects uterus to genital atrium. Vitellarium not evident. Tegumental surface covered by very thin papillae.

**Figure 3.**
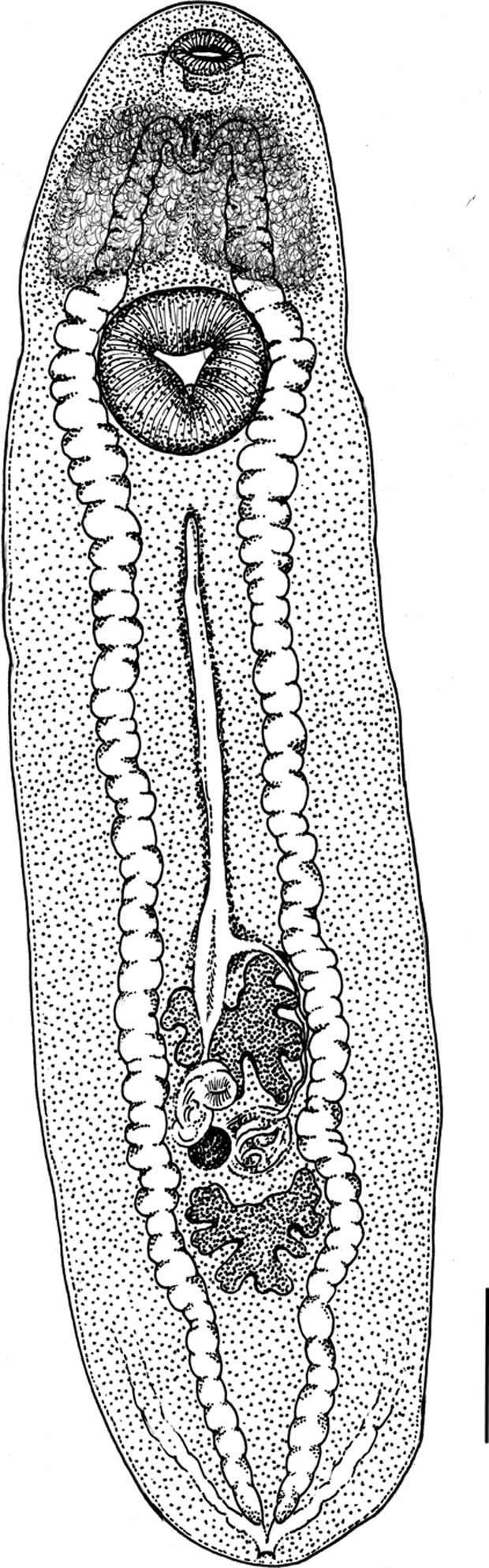
Line drawing of *Clinostomum phalacrocoracis* metacercaria. Scale bar = 1 mm.

The ITS sequence was 1030 bp: 584 bp belonged to ITS1, 159 bp to 5.8S and 287 bp to ITS2 rRNA. The BLAST analysis gave 100% identity with *C. phalacrocoracis* (FJ609422-FJ609423 [8]), 98.8% with *C. cutaneum* (FJ609421-GQ339114 [8]), 97.2% with *C. complanatum* (JF718629, [3]), 94% with *C. marginatum* (JF718634, [3]) and 92.6% with *C. tataxumui* (JX631065 [20]). The multiple alignment of our ITS rDNA sequences together with the *Clinostomum* sequences retrieved from GenBank revealed distances ranging from 1.2% to 7.3% over 867 positions. The distance analysis of the eight COI mtDNA sequences (605 bp) obtained in this study showed that all were identical to each other except for one (sample eight) showing three transitions (1 A/G and 2 T/C). The pairwise distance, calculated by including the *Clinostomum* COI sequences retrieved from GenBank and BOLDSYSTEMS (www.barcodinglife.org), ranged from 0.2% to 22.8% over 444 positions. It was not possible to BLAST the COI sequences of our *C. phalacrocoracis* as only *C. complanatum*, *C. marginatum* and *C. tataxumui* COI sequences were available for comparison.

**Figure 4.**
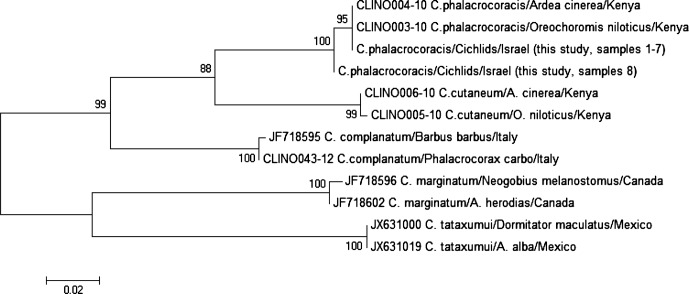
Phylogeny based on the Maximum Likelihood method using COI mtDNA sequences. The numbers correspond to the percentage of bootstrap support values derived from 1000 replicates. Samples 1–7 GB accession numbers KJ786967-KJ786969, KJ786971-KJ786974. Sample 8 GB accession number KJ786970.

The ML trees, utilizing both molecular markers, display the same topology (ITS tree not reported) and show two clearly separate clades, one formed by the Palearctic species, *C. complanatum*, that clusters as a sister species to *C. phalacrocoracis* + *C. cutaneum*, and the other by *C. marginatum* and *C. tataxumui*, the Nearctic and Neotropical species, respectively (Fig. 4). All sequences obtained during this study are deposited in GenBank under the accession numbers KJ786967-KJ786974 (COI) and KJ786975-KJ7869882 (ITS).

## Discussion

Three *Clinostomum* species have been reported so far in fish from Lake Kinneret, namely *C. cutaneum* [7, 15], *C. complanatum* [6, 15] and *C. tilapiae* [7, 27]. In this study, we describe metacercariae of *C. phalacrocoracis* for the first time from wild cichlids in Lake Kinneret. Dubois (1930) [4] originally described the adult stage of *Clinostomum phalacrocoracis* in *Phalacrocorax levaillanti* L. from Angola. Later, Ukoli [26] reported this species in African darter (*Anhinga rufa rufa*) in Ghana, Peirce and Din [18] reported it in *Pelecanus* spp. from Uganda, and Tendeiro et al. [25] in *P. onocrotalus* from Mozambique.

Kabunda and Sommerville [10] described metacercariae of *Clinostomum* sp. in tilapias (*Oreochromis* spp.) captured in Zaire showing morphological similarities to *C. phalacrocoracis* and *C. giganticum*. Based on our results we can state that the metacercaria described by Kabunda and Sommerville [10] and our specimens are *C. phalacrocoracis*.

The morphological characters of our specimens are consistent with the metacercaria of Kabunda and Sommerville [10] except the anterior testis, which we observed to be consistently fan-shaped, not saddle-shaped, as reported by these authors. This may be related to differences in metacercarial maturity, as stated by Ukoli [26], who reported less digitation in the testes of younger specimens. In 10 of 15 common metrics, including length, the specimens we collected were smaller than those of Kabunda and Sommerville [10], possibly due to different development or contraction of the body of our specimens.

These authors [10] also noted remarkable similarity between their metacercaria and *C. giganticum* described by Agarwal [1] in *Ophiocephalus punctatus* from India, but based on our observations the two species are different in the position of the genital pore, which is at the right posterior margin of the anterior testis between the lobes in *C. phalacrocoracis*, while it is at the level of the equator of the anterior testis in *C. giganticum.*

Concerning the other species described in Israel, at the metacercarial stage, the morphological characters observed in our specimens differ consistently from *C. cutaneum*, which has a unique Y-shaped uterus, the genital complex located in the middle part of the body and the genital pore opening close to the anterior margin of the anterior testis. Our specimens differ from *C. tilapiae* in the position of the genital complex, which is in the middle third of the body, the triangular testes, and the genital pore opening at the right posterior margin of the anterior testis, and from *C. complanatum* in the position of the genital pore and the shape of the testes.

In addition to the morphological characters, the molecular analysis carried out on the ITS rDNA region and COI mtDNA gene confirmed that our specimens belong to the species *C. phalacrocoracis*, displaying 100% identity in the ITS rDNA and the same nucleotide composition in COI (except sample eight with three nucleotide differences), as the same species collected by Gustinelli et al. [8] in tilapias and herons from Kenya.

The few detailed morphological descriptions of *C. phalacrocoracis* are from the African continent [4, 10, 23, 25, 26] together with a SEM description by Shaheen et al. [22]. Furthermore, several papers published afterward in Africa reported unidentified metacercariae of *Clinostomum* in different tilapia species [e.g. 2, 28, 29] but in all cases without any morphological and/or molecular description.

In addition to the presence of the fish second intermediate hosts, the completion of the life cycle of *C. phalacrocoracis* in the Lake Kinneret environment is reliant on the presence of its other hosts. Two snail species have been described in this area as the first intermediate host of *Clinostomum* spp., i.e. *Bulinus truncatus* and *Lymnaea auricularia* [7, 14, 17, 27]. However, in recent years these two species have not been reported in the lake, probably because of a decline in the lake water level [5]; we can speculate that the changes in snail populations influenced the disappearance of the *Clinostomum* species previously recorded and the emergence of *C. phalacrocoracis* in fish of Lake Kinneret, due to the involvement of other mollusc species in its life cycle [9]. Concerning the definitive host, the newly established dense vegetation in the drained area and waterfront has helped to increase the numbers of piscivorous birds [5, 14, 17]. Moreover, Lake Kinneret is located in the Rift Valley, a major seasonal bird migration route between northern Europe and Africa [5].

This description represents the second report of a *Clinostomum* species recorded in both Africa and Israel, after *C. cutaneum* [8, 15]. Moreover, Paperna [14] stated that water bodies in the Jordan River system throughout the Nile to Rift Valley lakes shared snail species and hosted similar fish.

In this study, we report for the first time *C. phalacrocoracis* metacercariae from cichlid fingerlings caught in Lake Kinneret, Israel, providing a morphological redescription of the metacercarial stage together with molecular data supporting its identification to the species level.
